# Heritability of education rises with intergenerational mobility

**DOI:** 10.1073/pnas.1912998116

**Published:** 2019-12-02

**Authors:** Per Engzell, Felix C. Tropf

**Affiliations:** ^a^Nuffield College, University of Oxford, Oxford OX1 1NF, United Kingdom;; ^b^Leverhulme Centre for Demographic Science, University of Oxford, Oxford OX1 1JD, United Kingdom;; ^c^Swedish Institute for Social Research, Stockholm University, 106 91 Stockholm, Sweden;; ^d^Laboratoire de Sociologie Quantitative, École Nationale de la Statistique et de l’Administration Économique, 99120 Palaiseau, France;; ^e^Department of Sociology, Center for Research in Economics and Statistics, 99120 Palaiseau, France

**Keywords:** educational attainment, intergenerational mobility, heritability

## Abstract

As an indicator of educational opportunity, social scientists have studied intergenerational mobility—the degree to which children’s attainment depends on that of their parents—and how it varies across place or time. We combine this research with behavior genetics to show that societal variation in mobility is rooted in family advantages that siblings share over and above genetic transmission. In societies with high intergenerational mobility, less variance in educational attainment is attributable to the shared sibling environment. Variance due to genetic factors is largely constant, but its share as a part of total variance, heritability, rises with mobility. Our results suggest that environmental differences underlie variation in intergenerational mobility, and that there is no tension between egalitarian policies and the realization of individual genetic potential.

Intergenerational education mobility—how strongly educational attainment persists from parent to child—is commonly used to indicate societies’ degree of openness or equality of opportunity (1, 2). A limitation of this literature is that it often is silent on the channels of transmission. Yet, we may view genetic transmission differently from other advantages such as parents’ ability to pay for good neighborhoods, schools, or access to college (3, 4). Insofar as genetic factors capture relevant abilities, their influence is consistent with meritocratic norms (5–7). Such norms can be motivated on grounds of efficiency, as a society’s viability depends on its ability to attract competent leaders and innovators (8, 9).

In other words, it matters not only whether education is inherited, but also how (3). One approach to the “how” question comes from behavior genetics (10). By comparing outcomes for family members with varying degree of genetic resemblance—typically, twins—we can partition variance in an outcome to that attributable to genetic factors (heritability, $h^{2}$), shared sibling environment ($c^{2}$), and idiosyncratic factors ($e^{2}$) (11). While such studies potentially tell us much about the distribution of opportunities, societal comparison has rarely been central to them. In this study, we ask: Where intergenerational mobility is higher, does the balance of “nature” and “nurture” in educational attainment differ?

## Results

### Intergenerational Mobility.

**Fig. 1.**
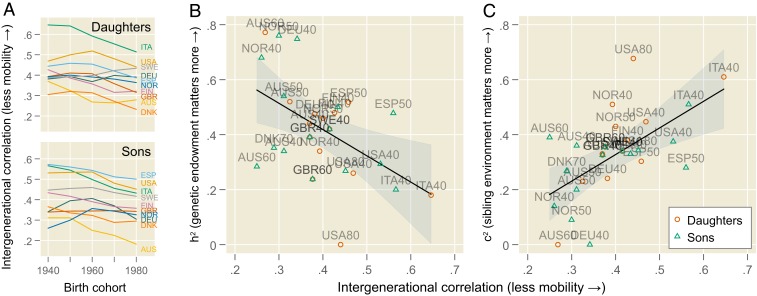
Genetic and environmental influences on educational attainment. (*A*) Trends in intergenerational mobility across 10 countries: Australia (AUS), Denmark (DNK), Finland (FIN), Germany (DEU), Italy (ITA), Norway (NOR), Spain (ESP), Sweden (SWE), United Kingdom (GBR), and United States (USA). (*B* and *C*) Association of intergenerational mobility with heritability ($h^{2}$) and shared environmental influences ($c^{2}$). Superimposed lines show the least-squares line of best fit, with 95% confidence intervals indicated by shaded areas; marker labels encode the country and decade of birth for each cohort.

Fig. 1*A* plots the parent–offspring correlation in years of schooling for children born in the 1940s to 1980s in 10 countries for which genetic data are available. A lower correlation implies more intergenerational mobility. These data, from the World Bank, confirm previous research: Educational mobility increased in the past and is higher in northern Europe and Australia—places with liberal welfare states—than in the United States or southern Europe (2, 12).

### Behavior Genetics Estimates.

In Fig. 1 *B* and *C*, we link intergenerational correlations with genetic data from twin studies (11). On the vertical axes, $h^{2}$ (Fig. 1*B*) tells us to what extent differences in educational attainment result from the genetic lottery, while $c^{2}$ (Fig. 1*C*) indicates the importance of other family background factors. In societies with high mobility—that is, a lower intergenerational correlation—the relative explanatory power of genetic factors ($h^{2}$) is stronger, while that of shared sibling environment ($c^{2}$) is weaker. Table 1 shows that this result remains when controlling for gender and adjusting standard errors for correlation between male and female subsamples. The residual component $e^{2}$ is largely unrelated to mobility.

**Table 1.** Correlation of standardized and destandardized variance components with the intergenerational correlation

	Standardized			Destandardized		
	$h^{2}$	$c^{2}$	$e^{2}$	$h^{2\prime }$	$c^{2\prime }$	$e^{2\prime }$
Coefficient	−0.512	0.574	−0.011	−0.056	0.726	0.342
Robust SE	0.142	0.121	0.179	0.284	0.147	0.207
*t* statistic	−3.61	4.74	−0.06	−0.20	4.94	1.65
P>|t|	0.003	0.000	0.951	0.848	0.000	0.121

The table shows correlations (standardized *β*) between *r*_IG_ and standardized and destandardized versions of *h*^2^, *c*^2^, and *e*^2^, including a fixed effect for gender. Standard errors are clustered for each birth cohort within a country (15 clusters).

### Absolute and Relative Variance.

That relative genetic influence $h^{2}$ is higher in more mobile societies could mean 2 things. Either 1) environmentally induced (and thereby total) variance is decreasing and/or 2) a lucky genetic draw is reaping higher rewards in absolute terms. If possibility 2, structural inequality may simply be replaced by genetic inequality, which could be seen as no less troubling. To test this, we destandardize $h^{2},c^{2},e^{2}$ by total population variance (Table 1). Doing so supports possibility 1 over possibility 2: the prevailing result is that high mobility goes together with a lower absolute influence of the shared environment, $c^{2\prime }$. In other words, $h^{2}$ and mobility correlate because mobility reduces total variance while genetic variance remains constant, not because genes matter more in an absolute sense.

## Discussion

Our results indicate that social mobility is improved by reducing social inheritance, a process that brings genetic influences to the fore. Besides their general interest, these findings speak to several academic debates. In social mobility research, there has been controversy about the extent to which policy can achieve lasting change. Critics have seen political attempts to increase mobility as either inadequate (13) or futile, adding the assumption that inheritance is largely genetic in origin (14). Our study challenges these views by showing that societal variation in mobility is not only substantial but firmly rooted in the removal of environmental barriers.

Meanwhile, geneticists have surmised that more ample opportunities may translate into a higher $h^{2}$ for developmental outcomes (15, 16). Based on this, it stands to reason that $h^{2}$ should be higher in societies where education policy promotes social mobility (17). Yet, comparative evidence has been lacking, and the canonical reference is to a study of Norwegian cohorts over time (18). A separate literature examines differences in $h^{2}$ between socioeconomic strata within a society, focusing mostly on IQ, but with mixed results (16, 17).

Our study contributes to these debates using large-scale cross-national data on an outcome of high policy relevance. Nevertheless, educational attainment is an incomplete proxy for social standing, and our results might have looked different with access to comparable data on income, wealth, or occupational prestige. Notably, research typically finds larger environmental components for economic outcomes (19). The intergenerational correlation is also distinct from causal effects of parental education, or the sibling correlation (20).

Lastly, it remains debatable whether genetic advantages should be seen as less troubling than socially inherited ones. While social inheritance may be easier to subvert, we should want to compensate for genetic bad luck when we can (21). Research using molecular genetic data can shed further light on the mechanisms underlying genetic influence (10, 22). Such research tells us that individual genetic variation contributes to social mobility and not just inheritance (23). More work in this vein will help grow the evidence base for policies aimed at ameliorating disadvantage.

## Materials and Methods

### Intergenerational Mobility.

Estimates are from the Global Database on Intergenerational Mobility (GDIM) (24) compiled by the World Bank (2) from various sample surveys. The main respondent is the daughter or son who also reports about the parents. We use the correlation between child ($y_{c}$) and parent ($y_{p}$) years of schooling,

$$r_{IG}=Corr\left(y_{c},y_{p}\right)=\frac{Cov\left(y_{c},y_{p}\right)}{\sqrt{Var\left(y_{c}\right)}\sqrt{Var\left(y_{p}\right)}},$$ [1]

for the parent with highest education. We smooth country trends by averaging the value for each cohort with the adjacent ones.

### Behavior Genetics Estimates.

Genetic variance components are from a metaanalysis by Branigan et al. (11). The $h^{2}$ (heritability) and $c^{2}$ (shared environmental variance) are estimated from the correlation in educational attainment among identical ($r_{MZ}$) and fraternal ($r_{DZ}$) twin pairs,

$$h^{2}=2\times \left(r_{MZ}-r_{DZ}\right),$$ [2]

$$c^{2}=r_{MZ}-h^{2},$$ [3]

$$e^{2}=1-h^{2}-c^{2},$$ [4]

where $e^{2}$ is a residual component reflecting nonshared environment and measurement error. To compare absolute and relative variances, we destandardize the components,

$$h^{2\prime }=h^{2}\cdot Var\left(y_{c}\right),$$ [5]

and do likewise for $c^{2}$ and $e^{2}$. Total population variance $Var\left(y_{c}\right)$ is estimated from the GDIM.

Twin studies make some strong model assumptions such as no genetic parental assortative mating, no gene–environment interaction, and that different types of twins share environmental influences effectively to the same extent. In our comparative study design, we mainly need to assume that potential model violations do not correlate with mobility patterns.

### Quality Control and Matching.

The GDIM spans cohorts born from the 1940s through the 1980s; genetic variance components span cohorts born throughout the century. We match each twin estimate to the GDIM based on country, gender, and the closest year of birth. Of the 34 estimates available, we exclude 2 cohorts that lack overlap in the GDIM data, and a further 3 that are from local or nonrepresentative samples in the United States. This leaves a total of 26 data points. The data contain a few negative variance estimates, which we set to zero.

### Data Availability.

Data and code to replicate all findings can be found at https://osf.io/c549j/. There we also show that our results are robust to analytical decisions taken, including the choice of parent, whether to smooth over cohorts, whether to exclude nonrepresentative US samples, whether to recode negative variance estimates, and how to account for clustering. Moreover, we confirm that our results are not driven by any one country, by repeating analyses excluding each.
